# The longitudinal progression of autonomic dysfunction in Parkinson's disease: A 7-year study

**DOI:** 10.3389/fneur.2023.1155669

**Published:** 2023-04-12

**Authors:** Charlotte B. Stewart, David Ledingham, Victoria K. Foster, Kirstie N. Anderson, Sahana Sathyanarayana, Debra Galley, Nicola Pavese, Jacopo Pasquini

**Affiliations:** ^1^Clinical Ageing Research Unit, Newcastle University, Campus for Ageing and Vitality, Newcastle upon Tyne, United Kingdom; ^2^Regional Sleep Service, Newcastle upon Tyne NHS Foundation Trust, Newcastle upon Tyne, United Kingdom; ^3^Department of Nuclear Medicine and PET Centre, Aarhus University Hospital, Aarhus, Denmark; ^4^Department of Clinical and Experimental Medicine, University of Pisa, Pisa, Italy

**Keywords:** autonomic dysfunction, SCOPA-AUT, PPMI (Parkinson's progression markers initiative), olfactory dysfunction, Parkinson's disease

## Abstract

**Background:**

Autonomic dysfunction, including gastrointestinal, cardiovascular, and urinary dysfunction, is often present in early Parkinson's Disease (PD). However, the knowledge of the longitudinal progression of these symptoms, and the connection between different autonomic domains, is limited. Furthermore, the relationship between the presence of autonomic symptoms in early-stage PD and olfactory dysfunction, a possible marker of central nervous system involvement, has not been fully investigated.

**Objectives:**

We aimed to investigate the occurrence and progression of autonomic dysfunction in recently diagnosed (< 2 years) untreated PD patients and determine any coexistence of symptoms in individual patients. We also investigated the relationship between autonomic symptoms, olfactory dysfunction, and motor impairment.

**Methods:**

Data were obtained from the Parkinson's Progression Markers Initiative (PPMI) database. Autonomic dysfunction was measured using the Scales for Outcomes in Parkinson's Disease (SCOPA-AUT). Symptom frequency and mean scores over 7 years were determined. The simultaneous occurrence of different autonomic symptoms was also examined. Finally, the relationships between SCOPA-AUT scores, olfactory dysfunction, and motor impairment were investigated using the University of Pennsylvania Smell Identification Test (UPSIT) and the Movement Disorder Society—Unified Parkinson's Disease Rating Scale (MDS-UPDRS), respectively.

**Results:**

Follow-up data were available for 7 years for 171 PD patients and for 5 years for 136 HCs. Mean SCOPA-AUT score increased significantly from baseline to the 7-year follow-up for each autonomic domain, except for female sexual dysfunction. Most patients reported three or more autonomic symptoms. Common clusters of symptoms were composed of combinations of gastrointestinal, urinary, thermoregulatory, and sexual dysfunction. At baseline, greater SCOPA-AUT total score was associated with lower UPSIT scores (*r* = −0.209, *p* = 0.006) and with greater total MDS-UDPRS III score (*r* = 0.218, *p* = 0.004).

**Conclusions:**

Autonomic dysfunction, often with coexistence of autonomic manifestations, is common in early PD and progressively worsens over the first 7 years of disease, suggesting that these symptoms should be addressed with appropriate treatments early in the disease. The association between greater autonomic dysfunction and greater olfactory impairment, coupled with the association with more severe motor scores at baseline, indicates that patients who show more severe autonomic dysfunction could also have more severe involvement of the central nervous system at the time of diagnosis.

## Introduction

Autonomic dysfunction is common in Parkinson's disease (PD) and poses a significant impact on patients' quality of life (1). It is increasingly recognized that autonomic symptoms, manifesting as gastrointestinal, urinary, cardiovascular, thermoregulatory, and pupillomotor dysfunction, can be present in the early stages of PD. A previous study by Stanković and colleagues found that 71% of early PD patients, classified as Hoehn and Yahr (H&Y) stage I, reported having autonomic symptoms (2). Autonomic dysfunction may also be a prodromal manifestation of PD, with early autonomic features predicting a faster rate of PD progression (3–5). Furthermore, pathological studies have shown the presence of alpha-synuclein deposition within the nuclei of autonomic plexi, which has been found to correlate with accelerated cell death in the autonomic nervous system (6, 7). However, the knowledge of how autonomic dysfunction progresses and the degree to which different autonomic symptoms cluster together, is limited (2).

Olfactory dysfunction is also a defining PD feature, and is often a prodromal manifestation (8, 9). Approximately 10% of subjects with idiopathic loss of smell receive a diagnosis of PD within 10 years (10). Olfactory dysfunction is associated with male sex and non-motor manifestations such as apathy (11) and cognitive dysfunction (12). However, the relationship between olfactory and autonomic dysfunction is unclear, and its investigation may be worthwhile in light of the proposed “brain-first” and “body-first” models of disease progression. Indeed, this hypothesis proposes two main routes of PD pathological progression, one following an ascending route originating from peripheral organs, e.g., the gut, and one following a descending route originating from the olfactory-amygdala complex (13, 14).

Therefore, in this study we investigated: (i) the longitudinal progression and coexistence of autonomic symptoms in early PD over a period of seven years and compared it to a cohort of healthy controls; (ii) the presence of olfactory dysfunction; (iii) the association between autonomic dysfunction, olfactory impairment, and motor manifestations in early PD (13).

## Methods

### Study population

PD patients and healthy controls (HC) were retrieved from the Parkinson's Progression Markers Initiative (PPMI) database. PPMI is an ongoing, multicentre clinical study investigating the longitudinal progression of PD. The study includes treatment-naïve PD subjects, with a disease duration of 2 years or less. Loss of dopaminergic neurons in the nigrostriatal tracts is confirmed in all subjects using dopamine transporter - single photon emission computed tomography (DaT-SPECT). HCs were all ≥30 years old and had no first-degree family members diagnosed with PD. HC diagnosed with PD at any time-point over the course of the study were excluded from analysis. A complete list of inclusion and exclusion criteria for the study can be found in the PPMI study protocol (https://www.ppmi-info.org/sites/default/files/docs/archives/Amendment-12.pdf). All participating PPMI sites received approval from an ethical standards committee prior to study initiation and written informed consent for research was obtained from all participants in the study.

Data for the analyses in this paper were accessed on the 2nd September 2022.

PD and HC subjects with seven years of follow-up data available were identified from the database. Participants who had missing data throughout the observation period were excluded from the analysis.

We identified 171 PD participants with complete datasets for 7-year analysis. In the HC group, 196 participants with baseline SCOPA-AUT data were available. Only a small number of participants had available data at year 7 (*n* = 23) or year 6 (*n* = 101), whereas 136 had available data for 5 years of follow-up. Therefore, we included only 5 years of follow-up data in the analysis of the HC population.

### Clinical assessment and demographics

Autonomic dysfunction was evaluated using the self-completed SCOPA-AUT questionnaire (15), assessing the following six autonomic domains, using their corresponding subscales: gastrointestinal (questions 1–7), urinary (questions 8–13), cardiovascular (questions 14–16), thermoregulatory (questions 17–18 and 20–21), pupillomotor (question 19) and sexual (questions 22–23 for males, and 24–25 for females). Total scores were calculated from the sum of all responses from questions 1–23 for male participants, and 1–21 and 24–25 for female participants. Each item score ranges from 0 to 3, based on the occurrence of specific autonomic symptoms; 0 (never), 1 (sometimes), 2 (regularly), and 3 (often).

To assess the frequency of autonomic symptoms, the percentage of subjects reporting a score > 0 for each item, representing individuals experiencing autonomic symptoms sometimes, regularly, and often, was calculated at each follow-up. The percentage of subjects reporting symptoms in a single autonomic domain (e.g., cardiovascular domain, questions 14–16) was determined by identifying subjects with a total domain score > 0.

The coexistence of autonomic symptoms in two or more domains was determined by analyzing the percentage of subjects reporting symptoms (SCOPA-AUT domain score > 0) in multiple domains. The percentage of subjects scoring above zero in one, two, three, four, five and all six autonomic domains, was determined. The data was also analyzed to assess whether there was any clustering of autonomic symptoms across PD participants. A cluster was defined by ≥ 10 individuals reporting a specific combination of symptoms.

Olfactory dysfunction was assessed with the University of Pennsylvania Smell Identification Test (UPSIT), and motor impairment with the Movement Disorder Society – Unified Parkinson's Disease Rating Scale (MDS-UPDRS). Published normative UPSIT scores were used to define normosmia, hyposmia, and anosmia (16).

### Statistical analysis

Baseline demographics and general clinical characteristics of PD patients and HCs were compared using the Chi-square test for categorical variables (e.g., gender) and Mann-Whitney test for continuous variables. To investigate the progression and differences between PD and HC in SCOPA-AUT total scores and sub-scores (gastrointestinal, urinary, cardiovascular, pupillomotor, thermoregulatory, male/female sexual dysfunction), two-way repeated measures ANOVA models were implemented for each dependent variable, and a grouping variable (HC or PD) was used as a factor. For each dependent variable, the Greenhouse-Geisser corrected overall model (group^*^assessment) was considered to evaluate the significance of the model. Univariate tests and Bonferroni-corrected pairwise comparisons based on estimated marginal means (EMMs) were used to test differences between PD and HC scores at each assessment. Multivariate tests and Bonferroni-corrected pairwise comparisons based on EMMs were used to test scores' differences between assessments in each group. Since data from year 5 to year 7 was present for PD participants only, one-way repeated measures ANOVA models were carried out to investigate the progression of SCOPA total score and sub-scores in such timeframe. To investigate statistical differences in the proportions of PD and HC reporting symptoms at baseline and each annual follow-up, Chi-square tests with Bonferroni *p*-value correction for multiple comparisons were used. To investigate the relationship between UPSIT, SCOPA-AUT and MDS-UPDRS III scores, Pearson correlation was carried out in PD subjects at baseline.

Statistical analysis was performed using SPSS Version 28 (IBM SPSS). Statistical significance level for hypothesis testing was set at *p* < 0.05, two-sided.

## Results

### Baseline demographics and clinical characteristics

**Figure 1:**
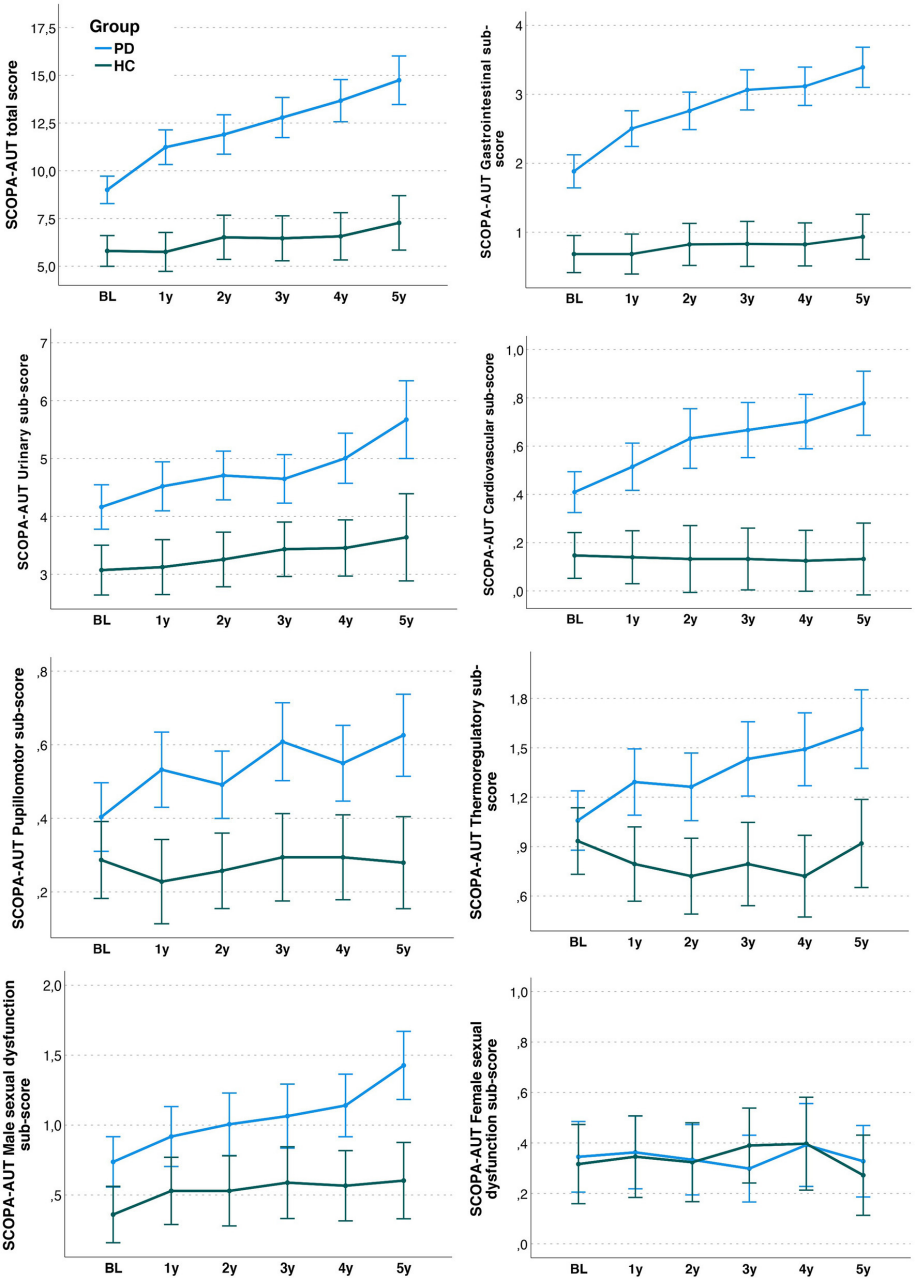
SCOPA-AUT total and sub-domain scores in Parkinson's disease (PD) and Healthy Controls (HC) over the first 5 years after baseline. Scores depicted in the panels are based on estimated marginal means (and 95% confidence intervals) from repeated-measures ANOVA models.

Gender proportions (χ2 = 2.823, *p* = 0.093) and age (*U* = 11705.5, *p* = 0.920) were not significantly different between PD and HC (Table 1). At baseline, PD patients had a significantly higher mean SCOPA-AUT total score and sub-scores for gastrointestinal, urinary, cardiovascular, and male sexual domains compared to HCs (all Bonferroni-corrected p_s_ < 0.01, from the repeated measures ANOVAs comparing baseline scores; Figure 1). Mean scores within the pupillomotor, thermoregulatory, and female-specific sexual domains, however, were not significantly different between patients and HCs at baseline.

**Table 1:** Baseline clinical and demographic features of Parkinson's Disease (PD) and healthy participants (HC).

	**Baseline information of PD subjects *N* = 171**	**Baseline information of HCs *N* = 136**	***p*-value**
Gender Number, Female/male (%)	51/120 (29.8/70.2)	53/83 (39.0/61.0)	
			0.093^a^
Age, years, mean (SD)	60.55 (9.81)	60.22 (11.27)	0.920^b^
Disease duration at enrolment, months, mean (SD)	6.62 (6.63)	-	-
Reported symptoms duration at enrolment, months, mean (SD)	49.28 (20.27)	-	-
MDS-UPDRS part III, mean (SD)	19.37 (8.56)	-	-
Hoehn and Yahr, median (range)	1 (1–3)	-	-
**SCOPA-AUT scores**			
Gastrointestinal	1.88 (± 1.95)	0.68 (± 0.98)	**<** **0.001**^**c**^
Urinary	4.16 (± 2.75)	3.07 (± 2.28)	**<** **0.001**^**c**^
Cardiovascular	0.41 (± 0.67)	0.15 (± 0.39)	**<** **0.001**^**c**^
Pupillomotor	0.40 (± 0.68)	0.29 (± 0.53)	0.102^c^
Thermoregulatory	1.06 (± 1.24)	0.93 (± 1.15)	0.366^c^
Sexual - male	0.74 (± 1.01)	0.36 (± 0.94)	**0.007** ^**c**^
Sexual - female	0.35 (± 1.35)	0.32 (± 0.81)	0.787^c^
TOTAL	9.00 (± 5.42)	5.80 (± 3.81)	< 0.001^c^

(^a^) chi-square test; (^b^) Mann-Whitney U test; (^c^) pairwise comparisons from repeated measures ANOVA models, Bonferroni-corrected *p*-values; MDS-UPDRS, Movement Disorders Society—Unified Parkinson's Disease Rating Scale; SCOPA-AUT, Scales for Outcomes in Parkinson's Disease-Autonomic Dysfunction; SD, Standard Deviation.

### Longitudinal progression of autonomic symptoms over 7 years

In PD, there was a significant increase in mean total SCOPA-AUT score from baseline to 5 years (baseline: EMM [SE]: 9.00 [0.37]; 5 years: EMM [SE] 14.74 [0.65]; F = 25.050, *p* < 0.001) and from 5 to 7 years (7 years: EMM [SE] 17.13 [0.74]; F = 9.294, *p* < 0.001). No significant increase was shown in HC (baseline: EMM [SE]: 5.81 [0.41]; 5 years: EMM [SE] 7.27 [0.73]; F = 1.667, *p* = 0.142). Figure 1 shows SCOPA-AUT total and sub-domain scores in PD and HC over the first 5 years after baseline.

In PD patients, SCOPA-AUT sub-scores significantly increased from baseline to the 5th year of follow-up in the gastrointestinal (F = 25.329, *p* < 0.001), urinary (F = 7.837, *p* < 0.001), cardiovascular (F = 7.540, *p* < 0.001), pupillomotor (F = 4.553, *p* < 0.001), thermoregulatory (F=5.232, *p* < 0.001), and male sexual dysfunction (F = 7.924, *p* < 0.001) domains. No change in the female sexual dysfunction domain score was found (F = 0.856, *p* = 0.511). No significant changes were found in sub-domains in HC.

At the 5 years follow up, SCOPA-AUT total score and all domains sub-scores, except female dysfunction, were significantly higher in PD compared to HC (all Bonferroni-corrected ps < 0.05; Figure 1). Thermoregulatory and pupillomotor dysfunction sub-scores, which were not different between PD and HC at baseline, became significantly different at first-year follow-up, and this difference remained significant thereafter (all Bonferroni-corrected p_s_ ≤ 0.001).

### Symptom frequencies

Urinary dysfunction was the most frequently reported symptom at baseline and during follow up for both patients and controls (Table 2). In controls, the most frequently reported urinary symptoms were increased frequency (69.9%) and nocturia (81.6%). The second-most common symptom domain in PD was gastrointestinal dysfunction. The comparison between groups showed that the percentage of PD subjects reporting gastrointestinal and cardiovascular symptoms were significantly greater than controls (*p* < 0.0001) at every time-point. Higher frequencies of symptoms were also identified in the PD group compared to the HC group, in the pupillomotor, thermoregulatory, and sexual domains, but these were not consistent over time. No differences were identified between the two groups in urinary dysfunction and female sexual dysfunction frequencies.

**Table 2:** Symptom frequencies from baseline to year 5 in HC, and to year 7 in PD.

**Number and percentage of subjects reporting symptoms in each domain**									
**Time-point**	**Baseline**			**Year-1**			**Year-2**		
**SCOPA-AUT domain**	**PD**	**HC**	***p***	**PD**	**HC**	***p***	**PD**	**HC**	***p***
Gastrointestinal	121 (71%)	56 (41%)	**< 0.0001**	142 (83%)	56 (41%)	**< 0.0001**	147 (86%)	57 (42%)	**< 0.0001**
Urinary	165 (96%)	125 (92%)	0.081	163 (95%)	125 (92%)	0.218	165 (96%)	126 (93%)	0.132
Cardiovascular	55 (32%)	18 (13%)	**< 0.0001**	61 (36%)	18 (13%)	**< 0.0001**	66 (39%)	14 (10%)	**< 0.0001**
Pupillomotor	54 (32%)	35 (26%)	0.262	67 (39%)	26 (19%)	**< 0.0001**	69 (40%)	32 (24%)	0.002
Thermoregulatory	99 (58%)	73 (54%)	0.459	105 (61%)	60 (44%)	0.003	102 (60%)	59 (43%)	0.005
Sexual	79 (46%)	45 (33%)	0.020	86 (50%)	54 (40%)	0.064	88 (51%)	47 (34%)	0.003
Male (*N* = 120; 83)	53 (44%)	22 (27%)	0.017	60 (50%)	31 (37%)	0.107	64 (53%)	27 (33%)	0.003
Female (*N* = 51; 53)	26 (51%)	22 (42%)	0.333	26 (51%)	22 (42%)	0.333	24 (47%)	20 (38%)	0.336
**Time-point**	**Year-3**			**Year-4**			**Year-5**		
**SCOPA-AUT domain**	**PD**	**HC**		**PD**	**HC**		**PD**	**HC**	
Gastrointestinal	151 (88%)	61 (45%)	**< 0.0001**	152 (89%)	63 (46%)	**< 0.0001**	155 (91%)	66 (49%)	**< 0.0001**
Urinary	163 (95%)	129 (95%)	0.850	164 (96%)	125 (92%)	0.139	164 (96%)	130 (96%)	0.891
Cardiovascular	74 (43%)	16 (12%)	**< 0.0001**	77 (45%)	15 (11%)	**< 0.0001**	77 (45%)	15 (11%)	**< 0.0001**
Pupillomotor	78 (46%)	34 (25%)	**< 0.0001**	70 (41%)	35 (26%)	0.005	74 (43%)	32 (24%)	**< 0.0001**
Thermoregulatory	109 (64%)	60 (44%)	**0.001**	107 (63%)	59 (43%)	**0.001**	105 (61%)	70 (51%)	0.081
Sexual	90 (53%)	59 (43%)	0.107	99 (58%)	54 (40%)	0.002	104 (61%)	55 (40%)	**< 0.0001**
Male (*N* = 120;83)	67 (56%)	33 (40%)	0.037	73 (61%)	32 (39%)	0.003	82 (68%)	36 (43%)	**< 0.0001**
Female (*N* = 51;53)	23 (45%)	25 (47%)	0.832	26 (45%)	21 (40%)	0.245	22 (43%)	19 (36%)	0.447
**Time-point**	**Year-6**			**Year-7**		
**SCOPA-AUT domain**	**PD**	**HC**	***p***	**PD**	**HC**	***p***
Gastrointestinal	158 (92%)	-	-	163 (95%)	-	-
Urinary	166 (97%)	-	-	165 (96%)	-	-
Cardiovascular	84 (49%)	-	-	85 (50%)	-	-
Pupillomotor	82 (48%)	-	-	81 (47%)	-	-
Thermoregulatory	112 (65%)	-	-	125 (73%)	-	-
Sexual	98 (57%)	-	-	98 (57%)	-	-
Male ( *N* = 120;83)	77 (64%)	-	-	74 (62%)	-	-
Female (*N* = 51;53)	21 (41%)	-	-	21 (41%)	-	-

Values represent the number of subjects with a score in each domain >0, with the percentage in parentheses based on the total number of subjects (171 PD; 136 HC). The total score for the sexual domain is divided further into specific scores for male and female, in which the percentage is based on the total number of male and female PD and HC subjects, appropriately. *P-*values comparing PD and HC cohorts are derived from Chi-square tests with Bonferroni correction for multiple comparisons (corrected *p*-value threshold = 0.001).

### Coexistence of autonomic symptoms

**Figure 2:**
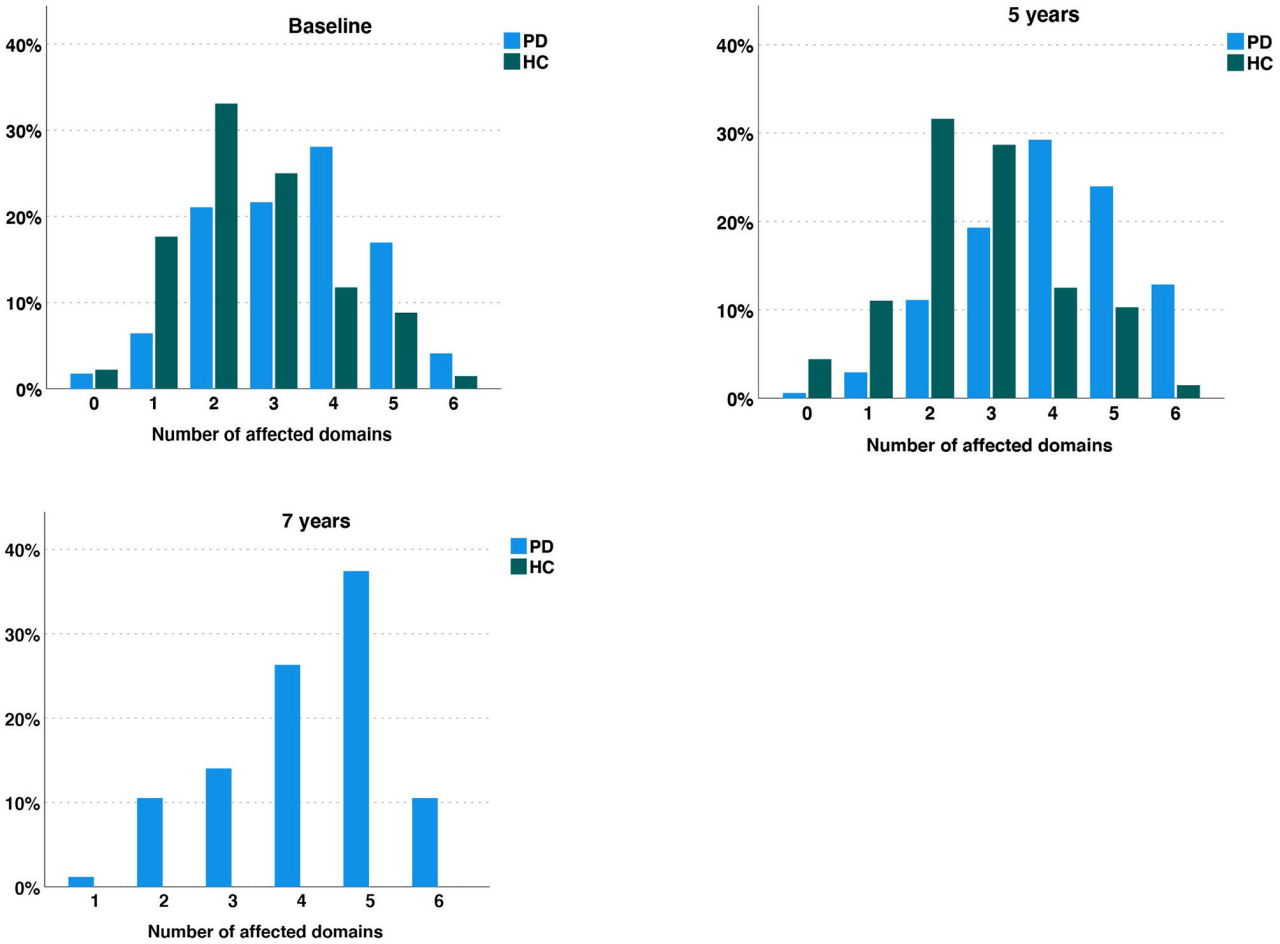
Diagram representing the percentage of Parkinson's disease (PD) patients and Healthy Controls (HC) reporting none, one, two, three, four five or all six autonomic symptoms over 5 years for both cohorts, and an additional 2 years in the PD group. Each column represents the percentage of participants in each group (PD or HC) with the indicated number of symptoms.

In the PD group, 2% reported no autonomic symptoms at baseline. Six percent of patients reported autonomic symptoms in one domain, 21% in two, 22% in three, 28% in four, 17% in five and 4% in all six domains (Figure 2). At 5-year follow-up, 1% of patients reported no autonomic symptoms, with 3% reporting autonomic symptoms in one domain, 11% reporting symptoms in two domains, 19% in three domains, 29% in four domains, 24% in five domains and 13% in all six domains. Finally, at seven years, all patients reported symptoms in at least one autonomic domain, with 1% experiencing symptoms in one domain only, 11% in two, 14% in three, 26% in four, 37% in five and 11% in all six.

**Figure 3:**
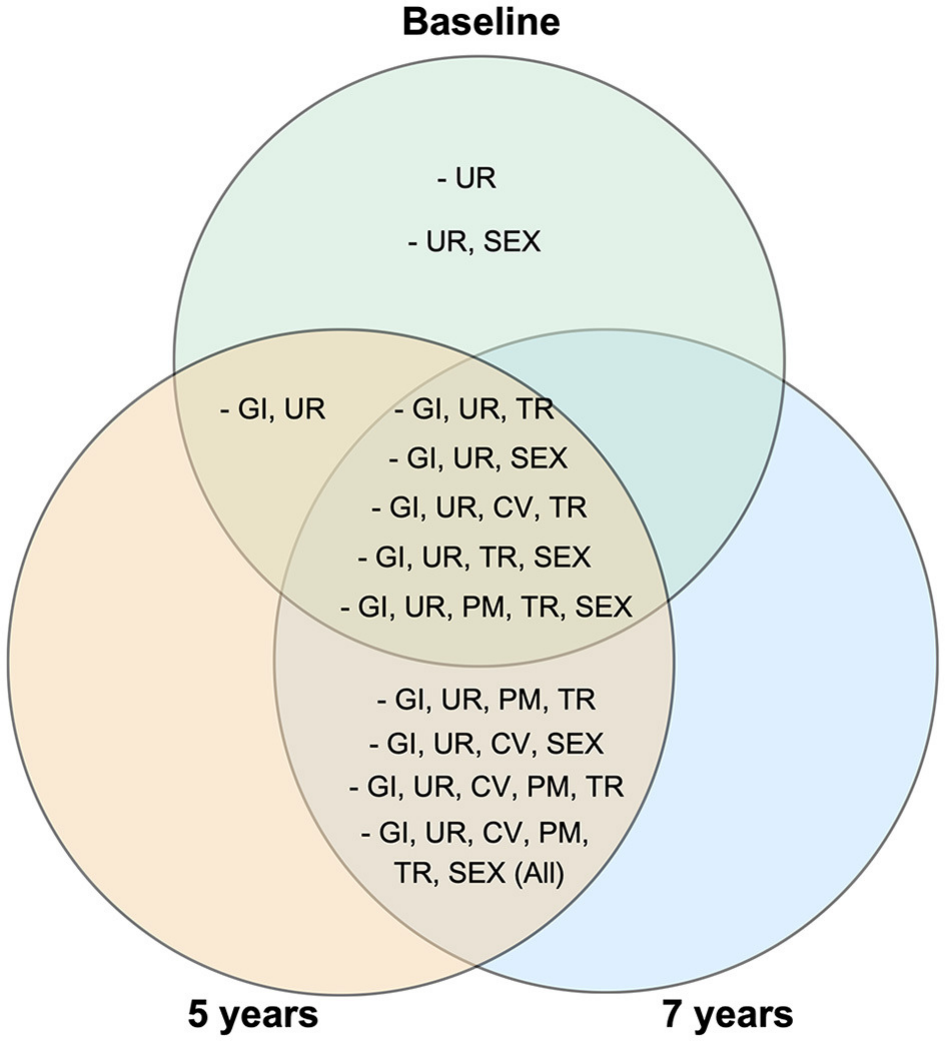
Venn diagram showing specific combinations of autonomic symptoms present in at least 10 Parkinson's disease participants at baseline, 5 years and 7 years follow ups. GI, gastrointestinal; UR, urinary; CV, cardiovascular; TR, thermoregulatory; PM, pupillomotor; SEX, sexual domain.

At baseline, eight clusters (i.e., ≥ 10 PD participants reporting a specific combination of symptoms) with common combinations of symptoms were identified: six of them contained gastrointestinal and urinary symptoms among others, while two of them included urinary symptoms only and urinary and sexual symptoms together. Interestingly, at 5 and 7 years, five clusters were overlapping with baseline (Figure 3). Ten clusters of symptoms were present at five years and nine at 7 years, and all of them included the gastrointestinal and urinary domains. Furthermore, at 5 and 7 years' follow up, a cluster of participants with symptoms in all domains was present.

### Association between autonomic, olfactory, and motor function

At baseline, 14 PD participants were normosmic, 95 hyposmic and 62 anosmic according to UPSIT normative scores. No differences were found between participants with a normal vs. abnormal score, or between hyposmic vs. anosmic participants, in baseline SCOPA-AUT total score, MDS-UPDRS part III total score and bradykinesia, rigidity and tremor sub-scores (Mann-Whitney U test, all p_s_ > 0.05).

Participants with baseline SCOPA-AUT total scores in the upper half of the scores' distribution (baseline SCOPA-AUT score median = 8) had significantly higher baseline MDS-UPDRS III total score compared to those in the lower half (median [interquartile range]: 22 [9] vs. 16.5 [9]; U = 2598.5, *p* = 0.002). Conversely, no significant differences were present in bradykinesia, rigidity, or tremor sub-scores between participants with SCOPA-AUT scores in the upper half of the distribution compared to those in the lower half.

**Figure 4:**
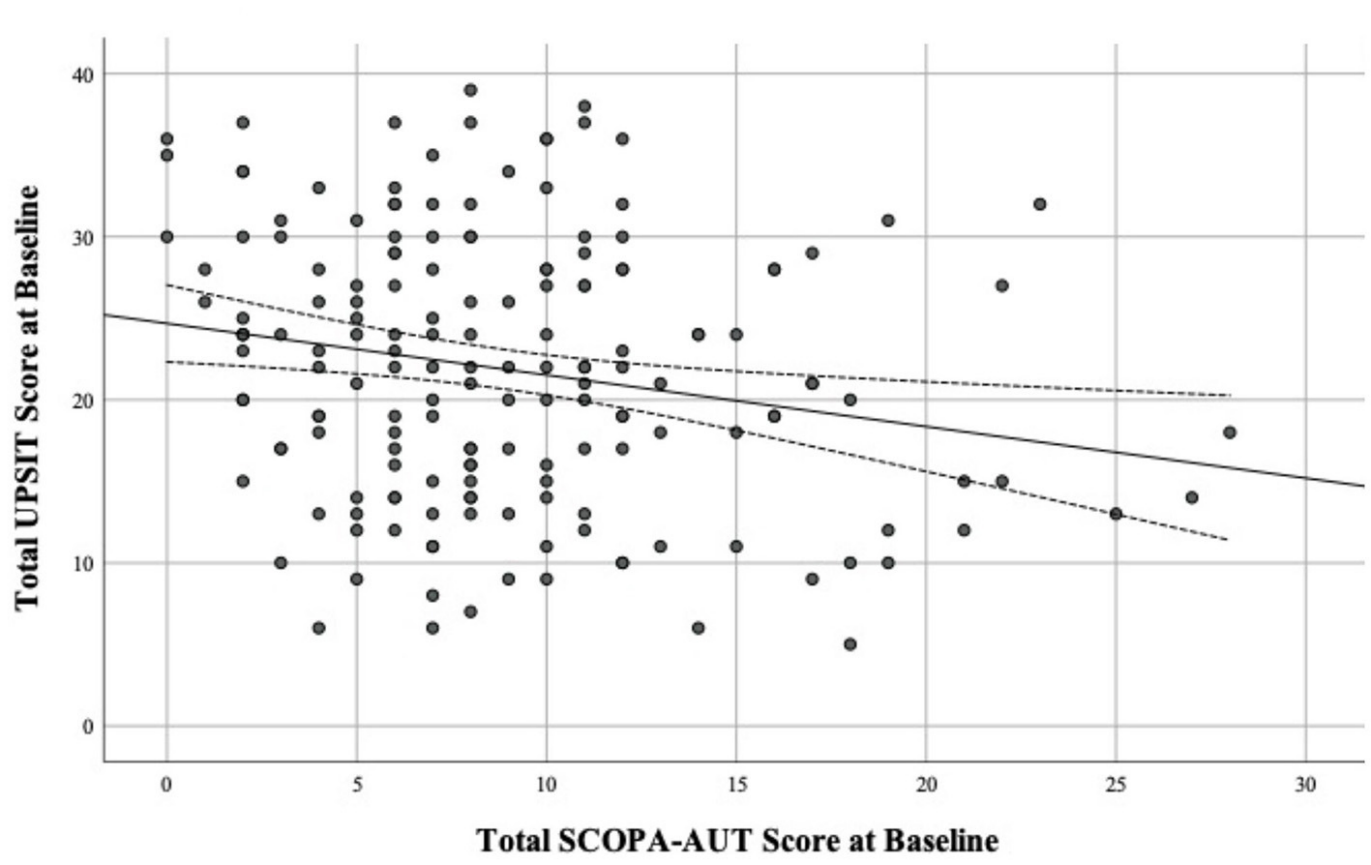
Scatter plot of total UPSIT score by total SCOPA-AUT score at Baseline in Parkinson's disease (PD), using Pearson correlation with 95% confidence intervals.

There was a significant inverse association between UPSIT and SCOPA-AUT total scores in PD (*r* = −0.209, *p* = 0.006, Figure 4) and HC subjects (*r* = −0.267, *p* = 0.002) at baseline. In PD, a significant direct association was also present between baseline MDS-UDPRS III score and SCOPA-AUT score (*r* = 0.218, *p* = 0.004).

## Discussion

Our study investigated the longitudinal progression of autonomic symptoms in a large, well characterized cohort of de novo PD patients over a period of seven years and followed the coexistence of autonomic symptoms over time to examine multi-domain clustering patterns. The association between autonomic, olfactory, and motor symptoms was also explored.

### Baseline demographics and clinical features

When comparing SCOPA-AUT scores between groups at baseline, we identified a significantly higher mean score in the gastrointestinal, urinary, cardiovascular, and sexual domains in PD patients compared to controls. However, while reported male sexual dysfunction symptoms were significantly greater in patients compared to controls, no significant difference was identified in female sexual dysfunction. Although under investigated, female sexual dysfunction has been reported in PD (17, 18). We believe our finding may be related to different factors, such as the early stage of the PD cohort that was studied and the fact that the SCOPA-AUT questionnaire, which contains only two specific questions for female sexual dysfunction, may be unable to identify differences with controls.

Conversely, no significant differences in mean scores were found between cohorts for thermoregulatory or pupillomotor domains. Taken together with a previous study (2), these results would suggest that pupillomotor dysfunction and possibly thermoregulatory dysfunction are mild and/or uncommon in the early stages of PD.

### Longitudinal assessment of autonomic symptoms over 7 years

Our study also investigated the longitudinal progression of autonomic dysfunction over a period of 7 years. To our knowledge this is the longest follow-up of autonomic dysfunction in a large cohort of early PD patients. Another strength of this study is that a complete dataset was available for PD subjects for 7 years, with no missing assessments, providing a realistic account of autonomic dysfunction in these patients over time.

We observed a significant increase in mean SCOPA-AUT score over time. This finding was consistent for all domains, excluding female sexual symptoms, which did not significantly increase over the observation period.

No significant increases in SCOPA-AUT total or sub-domains scores were shown in HC over the 5 years in which these subjects were observed.

One previous study with shorter follow-up (3 years) on 107 PD patients found increasing severity in urinary, gastrointestinal, cardiovascular domains, and sexual dysfunction. However, sexual dysfunction was not reported separately for males and females. Also, pupillomotor and thermoregulatory scores did not change over time (2). It should be noted that in that study the frequency of individual autonomic symptoms at baseline and the changes over time are smaller compared to the ones observed in our study. This may be due to factors related to the cohort that was assessed, consisting of participants with milder PD (H&Y stage I). Indeed, compared to the patients included in the current analysis from the PPMI cohort, their baseline mean MDS-UPDRS was 3.4 points lower and mean SCOPA-AUT total score 5.3 points lower.

Overall, our findings of worsening autonomic symptoms over time reinforce previous conclusions that autonomic dysfunction is associated with increasing PD severity and duration (2, 19–21) Furthermore, we add a detailed longitudinal analysis in all autonomic sub-domains over a long period of time, with a comparison between PD and HC over 5 years, and an additional follow up to a total of 7 years for PD.

### Frequency of individual autonomic symptoms over the 7-year period

Urinary dysfunction was the most reported autonomic symptom in individuals with PD at every time-point, affecting 95–97% of patients over the 7-year period. Interestingly, 92–96% of HCs also reported symptoms within the urinary domain, suggesting that urinary symptoms are also common in healthy controls (22). However, mean SCOPA-AUT scores in the urinary domain at baseline were significantly higher in PD than controls, indicating greater dysfunction in PD. In a previous large study of mid-stage PD patients (mean disease duration 10.5 years), greater scores in the urinary domain were found as well, compared to controls (23). Together with our findings, this suggests that urinary dysfunction may be present at the earliest stages of PD and worsens with disease progression.

Gastrointestinal dysfunction was the second most reported complaint among PD patients, with 71% experiencing symptoms at baseline, 91% at the 5-year follow-up, and 95% at 7 years. Within the gastrointestinal domain, constipation was found to be the most common symptom, followed by excessive drooling. Only 41–49% HCs reported these symptoms over the whole 5-year period. Our findings are in keeping with existing knowledge that PD patients frequently report gastrointestinal dysfunction both prior to the onset of motor symptoms and in early disease (23, 24). Of note there was a 24% increase in patients reporting gastrointestinal symptoms over the 7-year period, indicating that gastrointestinal dysfunction becomes more common with PD progression. A previous study conducted in a PD cohort with variable disease durations (mean 3.6 years and standard deviation 4.26) showed that greater right caudate dopaminergic deficit may be associated with greater gastrointestinal dysfunction (25).

Symptoms of cardiovascular dysfunction were reported by less than half of the PD patients at each assessment. However, at all assessments, the percentage of PD patients with these symptoms was significantly higher than the control population, suggesting a direct role of the neurodegenerative process in the occurrence of these symptoms.

Despite being one of the most common complaints at each assessment, the frequency of sexual dysfunction in males and females were not different between PD and controls until the 5-year follow-up assessment, when less than half (43%) of the male control population experienced sexual dysfunction compared to 62% of male PD patients. It is known that sexual symptoms, including erectile dysfunction, are commonly experienced by male PD patients (17), which occur in conjunction with other autonomic symptoms. Furthermore, reduced testosterone levels have also been shown in PD and this may contribute to such symptoms (26). It should also be acknowledged that scores of male sexual dysfunction were higher in PD than controls; therefore, although PD and HC showed similar frequencies, the severity was greater in PD.

Previous studies have identified sexual issues in females with PD, such as vaginal tightness and loss of libido (17, 18). The finding of more prominent sexual dysfunction in males than females with PD had also been reported in a previous small study of 34 patients with PD (27).

A previous study concluded that constipation, a drop in systolic blood pressure, and erectile dysfunction could identify PD 5 years before the diagnosis of the disease with a high sensitivity (4). Accordingly, at baseline (within 2 years of diagnosis) we identified that the largest difference in symptom frequencies between PD and HC subjects was in the gastrointestinal, cardiovascular, and male-specific sexual domains.

### Coexistence of autonomic symptoms in individual PD patients

We observed that the coexistence of autonomic symptoms in individual PD patients is very common, even in the first 2 years of disease, suggesting multi-organ involvement, which requires attention and appropriate management from the early stages of the disease. At baseline, only a minority of PD patients reported no autonomic dysfunction (3%) or a single symptom in any of the SCOPA-AUT domains (6%). Conversely, most of them (71%) reported symptoms in more than two domains and 4% reported problems in all six domains.

The percentage of patients experiencing multiple autonomic symptoms further increased over the 7-year follow-up, with all participants reporting at least one autonomic symptom at the last follow-up, and the percentage of patients reporting all six symptoms almost tripled to 11%.

The percentage of PD patients reporting five symptoms also increased from 17% to 37%, while the percentage of patients reporting autonomic symptoms in one or two domains dropped from 6% to 1% and from 21% to 11%, respectively.

Conversely, we did not observe a notable change in the proportions of HCs experiencing symptoms in multiple autonomic domains over the course of the 5-year follow-up period.

These findings further detail the progression of multiple autonomic symptoms from the early stages of PD (6) and extend the findings reported by Stanković and colleagues who also identified multi-domain dysfunction progressing over 3 years (2).

In our study, urinary symptoms were present in every PD cluster at baseline, 5 and 7 years, predominantly appearing with gastrointestinal, thermoregulatory, and sexual symptoms, suggesting these symptoms occur together in PD. Although to a lesser extent, cardiovascular and pupillomotor symptoms also appeared in conjunction with these symptoms. The clustering of these symptoms may be due to differential anatomical involvement of the peripheral autonomic nervous system, the dominant system innervating the organ (e.g., sympathetic or parasympathetic), and the residual innervation and function of the organ (28).

Although clustering of symptoms was also present in controls, the majority reported two or three symptoms, and only 1% experienced all six. It is probable that healthy controls will experience some autonomic symptoms due to, e.g., the normal process of ageing. It should also be acknowledged that our method of evaluating autonomic symptoms clustering does not consider severity and mean scores, which have been shown to be higher in the PD cohort (6).

### Association between autonomic and olfactory function in PD

At baseline, greater autonomic dysfunction was associated with both greater olfactory impairment and more severe motor scores. No differences were found in terms of motor manifestations between participants with and without olfactory dysfunction.

These findings may be indicative of a “clustering” of worse manifestations, i.e., patients with a more aggressive phenotype, as indicated in recent studies that have shown the possibility of subdividing PD populations in benign, intermediate and malignant subtypes (29, 30).

Furthermore, these findings should also be discussed in light of the hypothesis that the pathological PD process may progress in a bottom-up (body-first) or top-down (brain-first) fashion (8). Indeed, one could expect that PD patients with a “body-first” phenotype and predominant autonomic manifestations may have less olfactory impairment, and patients with a “brain-first” phenotype may have olfactory impairment and less autonomic dysfunction. However, in a “body-first” phenotype with autonomic symptoms, by the time motor symptoms arise, the pathological process may have already spread to cause olfactory dysfunction. Based on the available pathological evidence, it has been proposed that “body-first patients” might have a higher burden of cerebral Lewy body pathology (including in the olfactory bulbs) by the time PD becomes manifest, and this may, in turn, be associated with a higher degree of olfactory impairment (14). Conversely, in “brain-first patients” the involvement of the olfactory bulb may be more frequently unilateral, resulting in smaller olfactory impairment. In this context, the association between greater autonomic dysfunction and greater olfactory impairment may be driven by PD patients with a greater spread of pathology throughout the brain, a process that possibly started in the peripheral nervous system.

Finally, it may be hypothesized that the association between autonomic and olfactory dysfunction may be related to aging. Indeed, this same inverse correlation was also identified in the HC group. However, it should be noted that 65% of controls had normal olfactory function and overall low SCOPA-AUT scores, while PD participants were mostly hyposmic or anosmic and had significantly higher SCOPA-AUT scores than HC.

## Limitations

Several limitations should be acknowledged. Due to the lack of HCs with data at 6 and 7 years, we were unable to compare HCs and PD at the last two follow ups. We were also unable to control for PD medications, which may have effects on autonomic function. Similarly, concomitant conditions such as diabetes may also have influenced these symptoms, therefore further research may consider a full medical history of individual subjects to further improve the analysis, as well as uncover any associations with other pathologies.

We selected participants who had complete data over the entire follow up. This may have excluded more severe participants that were less able to attend the frequent PPMI visit schedule. Therefore, when interpreting the results of this study, it should be considered that the cohort might include a greater proportion of milder PD patients.

Our study focused on autonomic dysfunction. Other clinical and imaging data was not systematically included in the analysis. Further evaluation looking for other clinical and paraclinical associations of autonomic dysfunction may prove a useful avenue of future research.

It must also be acknowledged that the SCOPA-AUT does not include a threshold for allowing the clinical determination of dysautonomia in PD. In this study, the ability to determine whether scores reflect severe or mild dysfunction would have allowed a more apt analysis of autonomic function decline in PD. Furthermore, the subjective nature of the SCOPA-AUT opens the potential for underestimation or overestimation of autonomic symptoms, which could limit the significance of our results. Objective measures of autonomic dysfunction, alongside subjective SCOPA-AUT scores, would allow a more accurate analysis of autonomic function.

## Conclusions

Overall, our study provides novel insights into the progression of autonomic dysfunction in the first 7 years of idiopathic PD. A progressive increase in multi-domain dysfunction was identified over time. We also found that multiple autonomic symptoms in different organ domains cluster together in PD. Future studies investigating the progression of SCOPA-AUT score and multi-domain prevalence in PD in the later stages could lead to better understanding as to whether less frequently reported autonomic symptoms (e.g., cardiovascular and pupillomotor dysfunction) become more prevalent over time. Finally, at baseline higher autonomic dysfunction scores were associated with lower olfactory function scores, a finding that should be further investigated in future studies, considering the top-down and bottom-up models of PD pathology progression.

## Author's note

Members of Parkinson's Progression Markers Initiative (PPMI) are listed in the Supplementary material.

## Data availability statement

The data analysed in this study was obtained from the Parkinson's Progression Markers Initiative (PPMI) database (https://www.ppmi-info.org). Data used in the preparation of this article is available from the corresponding author upon request.

## Author contributions

CS: conception and design, acquisition of data, analysis, and interpretation write-up. JP: conception and design, analysis, and interpretation review. DL, KA, and SS: critical revision of article. VF and DG: critical revision of article and data collection. NP: critical revision of article and conception and design. All authors contributed to the article and approved the submitted version.
