# Corrigendum: The longitudinal progression of autonomic dysfunction in Parkinson's disease: a 7-year study

**DOI:** 10.3389/fneur.2023.1218633

**Published:** 2023-06-13

**Authors:** Charlotte B. Stewart, David Ledingham, Victoria K. Foster, Kirstie N. Anderson, Sahana Sathyanarayana, Debra Galley, Nicola Pavese, Jacopo Pasquini

**Affiliations:** ^1^Clinical Ageing Research Unit, Newcastle University, Campus for Ageing and Vitality, Newcastle upon Tyne, United Kingdom; ^2^Regional Sleep Service, Newcastle upon Tyne NHS Foundation Trust, Newcastle upon Tyne, United Kingdom; ^3^Department of Nuclear Medicine and PET Centre, Aarhus University Hospital, Aarhus, Denmark; ^4^Department of Clinical and Experimental Medicine, University of Pisa, Pisa, Italy

**Keywords:** autonomic dysfunction, SCOPA-AUT, PPMI (Parkinson's progression markers initiative), olfactory dysfunction, Parkinson's disease

In the published article, there was an error in the author list as published. The Parkinson's Progression Markers Initiative was erroneously excluded. The author list has now been updated.

In addition, an Author's note was missing from the published article. The updated Author's note appears below:

**Author's note**

Members of Parkinson's Progression Markers Initiative (PPMI) are listed in the Supplementary material.

In addition, a Supplementary material file listing the members of The Parkinson's Progression Markers Initiative was erroneously excluded from the publication. The Supplementary material has now been published alongside the original article.

In addition there was an error in the Acknowledgments statement as published. The date of data download, the full address of the PPMI database and the RRID number was missing. The updated Acknowledgments statement appears below.

**Acknowledgments**

Data used in the preparation of this article were obtained on September, 2nd 2022 from the Parkinson's Progression Markers Initiative (PPMI) database (www.ppmi-info.org/access-data-specimens/download-data), RRID:SCR_006431. For up-to-date information on the study, visit www.ppmi-info.org.

The authors apologize for these errors and state that this does not change the scientific conclusions of the article in any way. The original article has been updated.

