# Supplementary material for: The longitudinal progression of autonomic dysfunction in Parkinson's disease: A 7-year study
Source: Front Neurol. 2023 Apr 12;14:1155669. doi: 10.3389/fneur.2023.1155669 (PMC10130433; doi:10.3389/fneur.2023.1155669)
Supplement: Supplementary file 1 [file Data_Sheet_1.docx]

PPMI STUDY TEAMS/CORES/COLLABORATORS FOR PUBLICATIONS

Executive Steering Committee:

Kenneth Marek, MD1 (Principal Investigator); Caroline Tanner, MD, PhD9; Tanya Simuni, MD3; Andrew Siderowf, MD, MSCE12; Douglas Galasko, MD27; Lana Chahine, MD41; Christopher Coffey, PhD4; Kalpana Merchant, PhD61; Kathleen Poston, MD40; Roseanne Dobkin, PhD43; Tatiana Foroud, PhD15; Brit Mollenhauer, MD8; Dan Weintraub, MD12; Ethan Brown, MD9; Karl Kieburtz, MD, MPH23

Steering Committee:

Duygu Tosun-Turgut, PhD9; Werner Poewe, MD7; Susan Bressman, MD14; Jan Hammer15; Raymond James, RN22; Ekemini Riley, PhD42; John Seibyl, MD1; Leslie Shaw, PhD12; David Standaert, MD, PhD18; Sneha Mantri, MD, MS62; Nabila Dahodwala, MD12; Michael Schwarzschild47; Connie Marras45; Hubert Fernandez, MD25; Ira Shoulson, MD23; Helen Rowbotham2; Lucy Norcliffe- Kaufmann2; Paola Casalin11 and Claudia Trenkwalder, MD8

Michael J. Fox Foundation (Sponsor): Todd Sherer, PhD; Sohini Chowdhury; Mark Frasier, PhD; Jamie Eberling, PhD; Katie Kopil, PhD; Alyssa O’Grady; James Gibaldi, MSc; Maggie McGuire Kuhl; Leslie Kirsch, EdD

Study Cores, Committees and Related Studies: *(Include as applicable to the paper) Project Management Core:* Emily Flagg1
*Site Management Core:* Tanya Simuni, MD3; Bridget McMahon1
Strategy and Technical Operations: Craig Stanley1; Kim Fabrizio1

*Data Management Core:* Dixie Ecklund, MBA, MSN4; Trevis Huff4; Richard Peters4; Janel Fedler4 *Screening Core:* Tatiana Foroud, PhD15; Laura Heathers15; Christopher Hobbick15; Gena Antonopoulos15 *Imaging Core:* John Seibyl, MD1; Kathleen Poston, MD40
*Statistics Core*: Christopher Coffey, PhD4; Chelsea Caspell4; Michael Brumm, MS4
*Bioinformatics Core*: Arthur Toga, PhD10; Karen Crawford10
*Biorepository Core:* Tatiana Foroud, PhD15; Jan Hammer15
*Biologics Review Committee*: Brit Mollenhauer8; Doug Galasko27; Kalpana Merchant61
*Genetics Core:* Andrew Singleton, PhD13
*Pathology Core:* Tatiana Foroud, PhD15; Thomas Montine, MD, PhD40
*Found:* Caroline Tanner, MD PhD9
*PPMI Online:* Carlie Tanner, MD PhD9; Ethan Brown9; Lana Chahine41; Roseann Dobkin43; Monica Korell9

Site Investigators:

Ruth Schneider, MD23; Kelvin Chou, MD44; David Russell, MD, PhD1; Stewart Factor, DO16; Penelope Hogarth, MD17; Robert Hauser, MD, MBA19; Nabila Dahodwala, MD, MSc12; Marie H Saint-Hilaire, MD, FRCPC, FAAN22; David Shprecher, DO24; Hubert Fernandez, MD25; Kathrin Brockmann, MD26; Yen Tai, MD, PhD29; Paolo Barone, MD, PhD30; Stuart Isaacson, MD31; Alberto Espay, MD, MSc, FAAN, FANA32; Maria Jose Martí, MD, PhD34; Eduardo Tolosa MD, PhD34; Shu-Ching Hu, MD, PhD21; Douglas Galasko, MD27; Emile Moukheiber, MD28; Jean-Christophe Corvol, MD39; Nir Giladi, MD36; Javier Ruiz Martinez, MD, PhD35; Jan O. Aasly, MD37; Leonidas Stefanis, MD, PhD38; Karen Marder, MD MPH39 ; Arjun Tarakad, MD20; Connie Marras, MD, PhD, FRCP(C)45; Tiago Mestre, MD, PhD46; Aleksandar Videnovic, MD, MSc47; Rajesh Pahwa, MD48; Mark Lew, MD49; Holly Shill, MD50; Amy Amara, MD, PhD18; Charles Adler, MD, PhD51; Caroline Tanner, MD, PhD9; Susan Bressman, MD14; Tanya Simuni, MD3; Maureen Leehey, MD52; Giulietta Riboldi, MD53; Nikolaus McFarland, MD, PhD, FAAN54; Lana Chahine, MD41; Ron Postuma, MD, FRCPC55; Brit Mollenhauer, MD8; Werner Poewe, MD7; Zoltan Mari, MD56; Nicola Pavese, MD, PhD57; Michele Hu, MD, PhD58; Norbert Brüggemann, MD59; Christine Klein, MD, FEAN59; Bastiaan Bloem, MD, PhD60

Coordinators:

Anisha Singh, BS23; Angela Stovall, BS44; Julie Festa, BA1; Lianne Ramia, BS1; Katrina Wakeman, BS17; Karen Williams, BA, CCRP3;

Courtney Blair, MA18; Krista Specketer, BS21; Diana Willeke8; Jennifer Mule, BS25; Ella Hilt26; Shawnees Peacock, BS27; Kori Ribb, RN, BSN, CNRN28; Susan Ainscough, BA30; Lisbeth Pennente, BA31; Julia Brown, BS32; Christina Gruenwald, BS, CCRP32; Barbara Sommerfeld MSN, RN, CNRN16; Farah Kausar, PhD9; Alicia Garrido, MD34; Deborah Raymond, MS, CGC14; Ioana Croitoru35; Anne Grete Kristiansen37; Helen Mejia Santana, MA39; Anjana Singh, BS20; Danica Nogo, BS45; Shawna Reddie, BA46; Samantha Murphy, BS47; Lauren O’Brien48; Ashwini Ramachandran, MSc12; Fnu Madhuri, MS19; Daniel Freire, MS49; Farah Ismail, MBChB50; Raymond James, BS, RN22; Tom Osgood, BA, CCRP51; Heidi Friedeck, BS3; Jenny Frisendahl, BS52; Ying Liu, MD52; Caitlin Romano, BA53; Kelly Clark24; Kyle Rizer, BA54; Stephanie Carvalho39; Sherri Mosovsky, MPH41; Farah Sulaiman, MPH55; Dora Valent, MS7;

Raquel Lopes, BSN, MS29; Michelle Torreliza, AS56; Shira Paz, BS36; Victoria Kate Foster57; Madita Grümmer59; Myrthe Burgler, MA60; Sabine van Zundert, MS60; Christos Koros, MD, PhD38; Jamil Razzaque, MS58

1 Institute for Neurodegenerative Disorders, New Haven, CT 2 23andMe

3 Northwestern University, Chicago, IL

4 University of Iowa, Iowa City, IA

5 VectivBio AG

6 The Michael J. Fox Foundation for Parkinson’s Research, New York, NY 7 Innsbruck Medical University, Innsbruck, Austria

8 Paracelsus-Elena Klinik, Kassel, Germany

9 University of California, San Francisco, CA

10 Laboratory of Neuroimaging (LONI), University of Southern California 11 BioRep, Milan, Italy

12 University of Pennsylvania, Philadelphia, PA

13 National Institute on Aging, NIH, Bethesda, MD

14 Mount Sinai Beth Israel, New York, NY

15 Indiana University, Indianapolis, IN

16 Emory University of Medicine, Atlanta, GA

17 Oregon Health and Science University, Portland, OR 18 University of Alabama at Birmingham, Birmingham, AL 19 University of South Florida, Tampa, FL

20 Baylor College of Medicine, Houston, TX

21 University of Washington, Seattle, WA

22 Boston University, Boston, MA

23 University of Rochester, Rochester, NY

24 Banner Research Institute, Sun City, AZ

25 Cleveland Clinic, Cleveland, OH

26 University of Tuebingen, Tuebingen, Germany 27 University of California, San Diego, CA

28 Johns Hopkins University, Baltimore, MD

29 Imperial College of London, London, UK

30 University of Salerno, Salerno, Italy

31 Parkinson’s Disease and Movement Disorders Center, Boca Raton, FL 32 University of Cincinnati, Cincinnati, OH

34 Hospital Clinic of Barcelona, Barcelona, Spain

35 Hospital Universitario Donostia, San Sebastian, Spain

36 Tel Aviv Sourasky Medical Center, Tel Aviv, Israel

37 St. Olav’s University Hospital, Trondheim, Norway

38 National and Kapodistrian University of Athens, Athens, Greece

39 Columbia University Irving Medical Center, New York, NY

40 Stanford University, Stanford, CA

41 University of Pittsburgh, Pittsburgh, PA

42 Center for Strategy Philanthropy at Milken Institute, Washington D.C. 43 Rutgers University, New Brunswick, NJ

44 University of Michigan, Ann Arbor, MI

45 Toronto Western Hospital, Toronto, Canada

46 The Ottawa Hospital, Ottawa, Canada

47 Massachusetts General Hospital, Boston, MA

48 University of Kansas Medical Center, Kansas City, KS

49 University of Southern California, Los Angeles, CA 50 Barrow Neurological Institute, Phoenix, AZ

51 Mayo Clinic Arizona, Scottsdale, AZ

52 University of Colorado, Aurora, CO

53 NYU Langone Medical Center, New York, NY

54 University of Florida, Gainesville, FL

55 Montreal Neurological Institute and Hospital/McGill, Montreal, QC, Canada

56 Cleveland Clinic-Las Vegas Lou Ruvo Center for Brain Health, Las Vegas, NV 57 Clinical Ageing Research Unit, Newcastle, UK
58 John Radcliffe Hospital Oxford and Oxford University, Oxford, UK
59 Universität Lübeck, Luebeck, Germany

60 Radboud University, Nijmegen, Netherlands 61 TransThera Consulting
62 Duke University, Durham, NC
